# Pharmacological Modulation of Hemodynamics in Adult Zebrafish *In Vivo*

**DOI:** 10.1371/journal.pone.0150948

**Published:** 2016-03-11

**Authors:** Daniel Brönnimann, Tijana Djukic, Ramona Triet, Christian Dellenbach, Igor Saveljic, Michael Rieger, Stephan Rohr, Nenad Filipovic, Valentin Djonov

**Affiliations:** 1 Institute of Anatomy, University of Bern, Baltzerstrasse 2, 3012, Bern, Switzerland; 2 BioIRC R&D Bioengineering Center, Prvoslava Stojanovica 6, 34000, Kragujevac, Serbia; 3 Faculty of Engineering, University of Kragujevac, Sestre Janjic 6, 34000, Kragujevac, Serbia; 4 Institute of Physiology, University of Bern, Bühlplatz 5, 3012, Bern, Switzerland; 5 Harvard School of Public Health, Harvard University, Boston, United States of America; Mayo Clinic, UNITED STATES

## Abstract

**Introduction:**

Hemodynamic parameters in zebrafish receive increasing attention because of their important role in cardiovascular processes such as atherosclerosis, hematopoiesis, sprouting and intussusceptive angiogenesis. To study underlying mechanisms, the precise modulation of parameters like blood flow velocity or shear stress is centrally important. Questions related to blood flow have been addressed in the past in either embryonic or *ex vivo*-zebrafish models but little information is available for adult animals. Here we describe a pharmacological approach to modulate cardiac and hemodynamic parameters in adult zebrafish *in vivo*.

**Materials and Methods:**

Adult zebrafish were paralyzed and orally perfused with salt water. The drugs isoprenaline and sodium nitroprusside were directly applied with the perfusate, thus closely resembling the preferred method for drug delivery in zebrafish, namely within the water. Drug effects on the heart and on blood flow in the submental vein were studied using electrocardiograms, *in vivo*-microscopy and mathematical flow simulations.

**Results:**

Under control conditions, heart rate, blood flow velocity and shear stress varied less than ± 5%. Maximal chronotropic effects of isoprenaline were achieved at a concentration of 50 μmol/L, where it increased the heart rate by 22.6 ± 1.3% (*n = 4; p < 0*.*0001*). Blood flow velocity and shear stress in the submental vein were not significantly increased. Sodium nitroprusside at 1 mmol/L did not alter the heart rate but increased blood flow velocity by 110.46 ± 19.64% (*p = 0*.*01*) and shear stress by 117.96 ± 23.65% (*n = 9; p = 0*.*03*).

**Discussion:**

In this study, we demonstrate that cardiac and hemodynamic parameters in adult zebrafish can be efficiently modulated by isoprenaline and sodium nitroprusside. Together with the suitability of the zebrafish for *in vivo*-microscopy and genetic modifications, the methodology described permits studying biological processes that are dependent on hemodynamic alterations.

## Introduction

During the last decade, the zebrafish *(danio rerio)* has emerged as a broadly studied and well-documented model organism in basic research [1,2]. Externally developing zebrafish embryos are easily amenable to *in vivo*-observations [3] and genetic manipulations [4]. In particular, the assessment of hemodynamic parameters has received increasing attention because of their important role in cardiovascular processes. For example, shear stress was shown to be a major determinant of atherosclerosis, as plaques predominantly develop at side branches or at vessel bends, where blood flow is disturbed or generally low [5]. In zebrafish, *silent heart* embryos that lack a heartbeat and hence blood flow develop less hematopoietic stem cells [6]. Increased blood flow, local blood flow perturbations or the change from laminar to turbulent flow act as angiogenic triggers [7] and influence the vascular tone [8], sprouting [9,10] and intussusceptive angiogenesis [11,12]. Similar to atherosclerosis, intussusceptive angiogenesis was predominantly observed at sites of disturbed shear stress, i.e. branching points of blood vessels [11].

Cardiac function has been intensively studied in adult zebrafish *in vivo*, especially during cardiac regeneration [13,14]. However, data on how to effectively modulate cardiac and hemodynamic parameters are lacking. In zebrafish, modulations of the cardiac cycle in health and disease have been intensively studied *in vivo* in embryos [3,15,16] and *ex vivo* in isolated perfused hearts [17,18,19], with both models having their specific disadvantages. Embryonic zebrafish do not express all the necessary receptors present in adult zebrafish [20]. Vice versa, isolated hearts are devoid of neural and endocrine inputs and display a reduced performance with increasing time in culture [18] and consistently show lower heart rates than observed *in vivo* [17,19]. To avoid these disadvantages, we established a model to study the pharmacological modulation of cardiac and hemodynamic parameters in adult zebrafish *in vivo*.

In brief, our goal was to profile dynamically the effects of perfusion-administered cardio- and vasoactive drug solutions on heart rate, blood flow velocity and shear stress in adult zebrafish. Drug administration was performed on fish paralyzed with an intraperitoneal injection of μ-conotoxin GIIIB (μ-CTX), an inhibitor of Na_v_ channels [21]. Together with an oral saltwater perfusion, this method was proposed before for investigating the human long QT syndrome in zebrafish and is suitable for long-term cardiac investigations [21,22,23]. Hemodynamic changes were analyzed by comparing results to blood flow simulations *in silico*.

Taken together, we demonstrate how to pharmacologically interfere with the cardiovascular system of adult zebrafish and use the system to investigate the effects of Iso and SNP on heart rate, blood flow velocity and shear stress *in vivo*.

## Materials & Methods

All animal experiments conformed to the guidelines of the Swiss government and were approved by the Bundesamt für Veterinärwesen (BVET) under the license 25873.

### Animal maintenance

Wildtype (AB) zebrafish (green fluorescent endothelial cells), aged 10–14 months and weighing 250–300 mg were used for all experiments. The fish obtained from the EZRC (European Zebrafish Resource Center, Karlsruhe, Germany), were raised and maintained in system water at 28.5°C (conductivity: 500 μS, pH: 7.4) and a 14:10 h light and dark cycle. They were fed at least twice a day with live brine shrimp (Special Diets Services, Germany) or dry food (GM-300, Skretting, France).

### Immobilization

**Fig 1:**
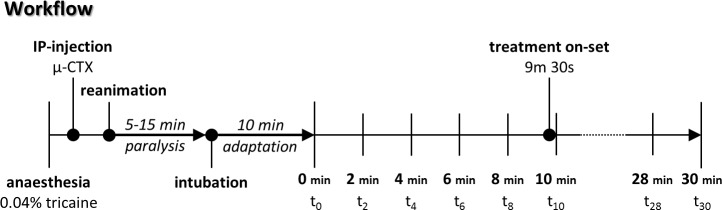
Workflow of routine investigations to measure hemodynamic and cardiac parameters *in vivo*. After an anesthesia with 0.04% tricaine, the fish were injected i.p. with 12 μl (1 nmol/L) of the paralyzing agent μ-conotoxin GIIIB (μ-CTX). After paralysis was observed, the fish was adapted to the oral perfusion for 10 min. Thereafter, baseline values have been acquired between t_0_ and t_8_. Drug solutions were orally administered with the perfusate at 9.5 min.

The immobilization procedure was adapted from Milan et al., 2006 [21] and depicted in Fig 1. First, the zebrafish were anesthetized with 0.04% tricaine (pH 7.4) and placed in a damp sponge. Paralysis was induced by intraperitoneal injection of 12 μl of 1 nmol/L μ-conotoxin GIIIB (μ-CTX) using a Hamilton syringe (Hamilton Analytics, Switzerland) equipped with a fine 33G-needle (Hamilton Analytics, Switzerland). Paralysis was complete within 5–15 min.

### Oral perfusion and drug administration

Paralyzed zebrafish were immediately intubated with an abraded 22G-needle (Terumo, Belgium) and orally perfused with 6 ml/min of system water by a peristaltic pump (220 min^-1^) connected to a micromanipulator (PRIOR, England). Control animals and experimental animals during baseline acquisition of the heart rate were perfused with system water. Drugs were dissolved in system water and administered via the oral perfusate. After the experiments, fish were immediately euthanized with a combination of rapid cooling and an overdose of tricaine [24].

### Chemicals

Isoprenaline hydrochloride (Iso), sodium nitroprusside dehydrate (SNP) and ethyl 3-aminobenzoate methanesulfonate (MS-222, tricaine) were purchased from Sigma Aldrich. Iso is a β1/β2-adrenoreceptor agonist [15,25,26], whereas sodium nitroprusside (SNP) is a potent nitric oxide donor. Nitric oxide plays a major role in regulating the vascular diameter, endothelial cell migration and angiogenesis [27,28,29,30]. The paralytic agent μ-conotoxin GIIIB was obtained from Alomone Labs, Israel.

### ECG recording

Differential electrocardiograms (ECG) were recorded with three 33G-needles (Hamilton Analytics, Switzerland) placed on the ventral midline between the two anal fins and left side of the heart. The reference electrode was connected to the perfusion needle. ECGs were acquired by a custom-made bio-signal acquisition system and analyzed using custom software (MATLAB version R2011b).

### Heart rate measurement

Optical determinations of heart rates were performed using a Leica stereomicroscope M205FA (Leica Microsystems, Switzerland) equipped with a Leica DFC365X camera (image acquisition software: Leica AF6000). Heart rates (HR) were extracted from 10 second-movies (15 frames per second) by optical evaluation followed by extrapolation to beats per minute.

### Blood Flow Measurements

Blood flow was assessed based on 3 second-movies of the submental blood vessel at 37 frames per second using a PCO.edge 5.5 sCMOS camera coupled to a Leica stereomicroscope M205FA.

### Blood Flow Simulations

A custom-made software PakF [31,32] based on the finite element method was used to simulate blood flow. This software is intended primarily for simulations of three-dimensional fluid flow in the arteries and is capable to numerically simulate plaque formation and progression [33,34,35]. Numerical simulations have been successfully applied for modeling many phenomena related to biology and medicine, including processes such as drug delivery or atherosclerosis formation, cancer progression or the motion of LDL-particles in the blood stream [29,36,37,38]. The geometrical domain was obtained from experimental images, as well as the velocity at the inlet. Velocity and shear stress were then calculated for the whole vascular segment using the mathematical model. A detailed description of the blood flow simulation procedure and all the used equations can be found in S1 Fig.

### Statistical Analyses

All statistical analyses were performed with GraphPad Prism v5.04. P-values of two-tailed paired t-tests were considered statistically significant if p < 0.05 (p: * <0.05, ** <0.01, *** <0.001). Values are given as mean ± standard deviation. The numerical data given in the main text of the results section is expressed as mean ± 95% confidence interval.

## Results

### Validation

**Fig 2:**
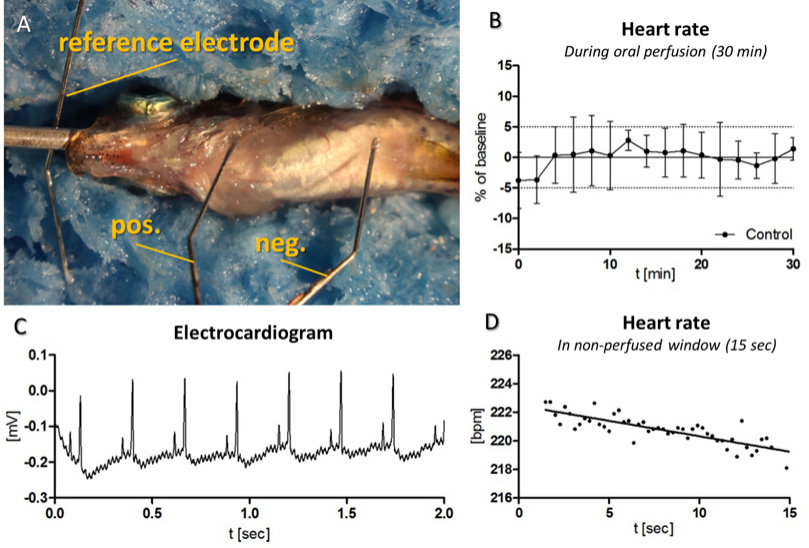
Electrocardiographic (ECG) measurements to validate heart rate (HR) stability during paralysis. (A) Placement of the electrodes for the ECG. The reference electrode was coupled to the perfusion needle. (B) Optical measurements of HR of non-treated zebrafish (n = 5). The HR remained constant with deviations of less than ±5%. (C) Example of an ECG recorded during stopped oral perfusion. (D) Representative image of plotted PP-intervals in a non-perfused window.

We first investigated the effects of paralysis and oral perfusion on the heart rate in adult zebrafish *in vivo*. The fish were paralyzed using μ-CTX, intubated and then orally perfused with system water using the protocol illustrated in Fig 1. An adaptation period of 10 min was introduced before the acquisition of baseline values to allow all parameters to stabilize. Motion artifacts due to the pulsatile oral perfusion were suppressed by stopping the peristaltic pump during data acquisition. Electrocardiograms (ECG) obtained from three electrodes (Fig 2A), were found to be regular and exhibited prominent P- and R-peaks (Fig 2C). Cessation of oral perfusion caused a linear (R^2^ = 0.92) decrease of the heart rate by 0.22 beats per second over 15s of stopped perfusion (Fig 2D). Therefore, heart rates (HR) were routinely determined within windows of no more than 10s. Measurements performed at 10s intervals for 30 min showed constant beat rates that deviated less than ± 5% from the mean beat rate (Fig 2B). A baseline of 9.5 min was introduced to abolish the late effects of the initial tricaine application before the intraperitoneal injections of μ-CTX.

### Heart rate dependence on isoprenaline

**Fig 3:**
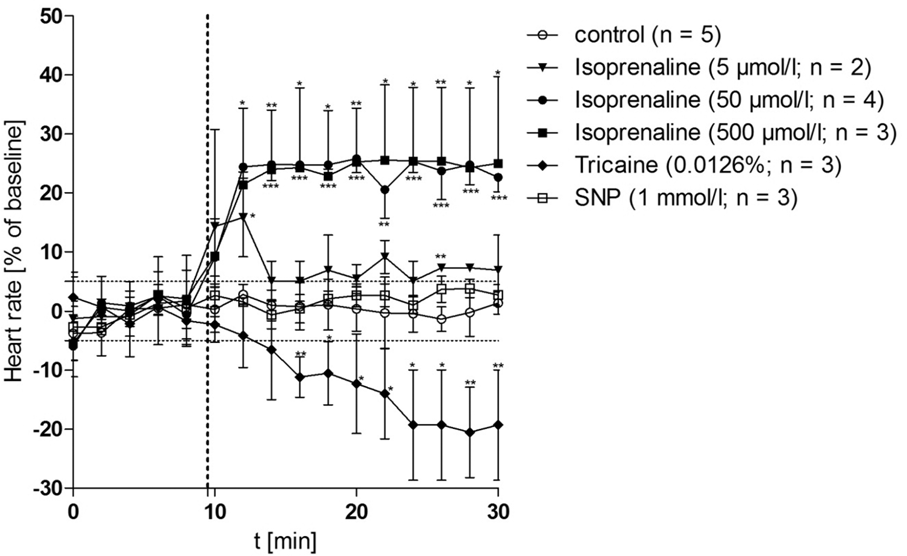
Optical measurement of heart rates in response to isoprenaline, tricaine and sodium nitroprusside (SNP) *in vivo*. The heart rates of the same fish were followed over a period of 30 min at intervals of 2 min. Values obtained between t_10_ and t_30_ are expressed as percentage of the baseline (t_0_-t_8_). Drug solutions were administered at 9.5 min (indicated by the dashed line).

We determined the effects of three concentrations of isoprenaline hydrochloride (Iso) on the heart rate *in vivo* (Fig 3). Iso caused a dose-dependent increase of HR with maximal effects seen at 50 μmol/L, where it increased HR by 22.6 ± 1.3% after 20 min of treatment. The onset of the chronotropic effect was rapid (estimated 44.7 ± 9.1 s (n = 4) to half maximal effect). In addition, we investigated the influence of 0.0126% tricaine, a concentration currently being used for long-term observations of zebrafish under anesthesia. This low dose of tricaine decreased the HR by 19.3 ± 5% after 20 min of treatment (n = 3). In contrast to Iso, tricaine action was characterized by a slow onset and allowed irregular movements of the fish when used without paralysis.

### Hemodynamic measurements in the submental vein

**Fig 4:**
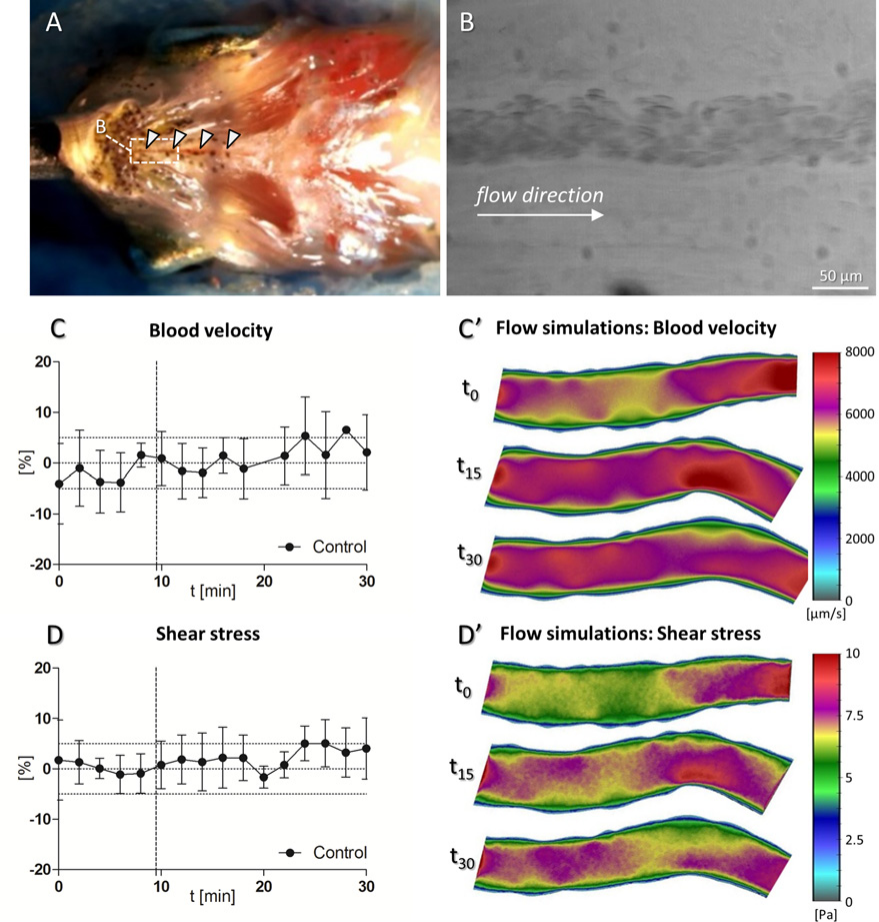
Blood velocity and shear stress measurements in the submental vein. (A) Ventral view of the head of an adult zebrafish. White arrowheads indicate the submental vein. (B) Still image of a 3 second-movie of the blood flow in the submental vein. (C+D) Blood velocity at the inlet and shear stress plotted in percent as deviation from baseline (t_0_-t_8_). (C’+D’) Simulations of blood velocity and shear stress performed at the time points designed in C+D.

Hemodynamic measurements were performed in the submental vein, whose diameter ranged from 40 μm to 70 μm (Fig 4B). To assess blood flow, 3 second-movies covering ~300 μm of the length of the vessel were acquired at 37 frames per second (fps). Based on these movies, we assessed variations in blood velocity (Fig 4C) and shear stress (Fig 4D) over a time period of 30 min. Both parameters remained largely constant with deviations not exceeding ±5%. Simulations of blood velocity and shear stress based on the anatomy of the vessel showed that the two parameters were homogeneously distributed within the same vascular section over time (Fig 4C’ and 4D’). Velocity and shear stress were increased both at vessel bends and at the inlets/outlets designated at the borders of the acquired movies.

### Sodium nitroprusside (SNP) affects blood velocity and shear stress

**Fig 5:**
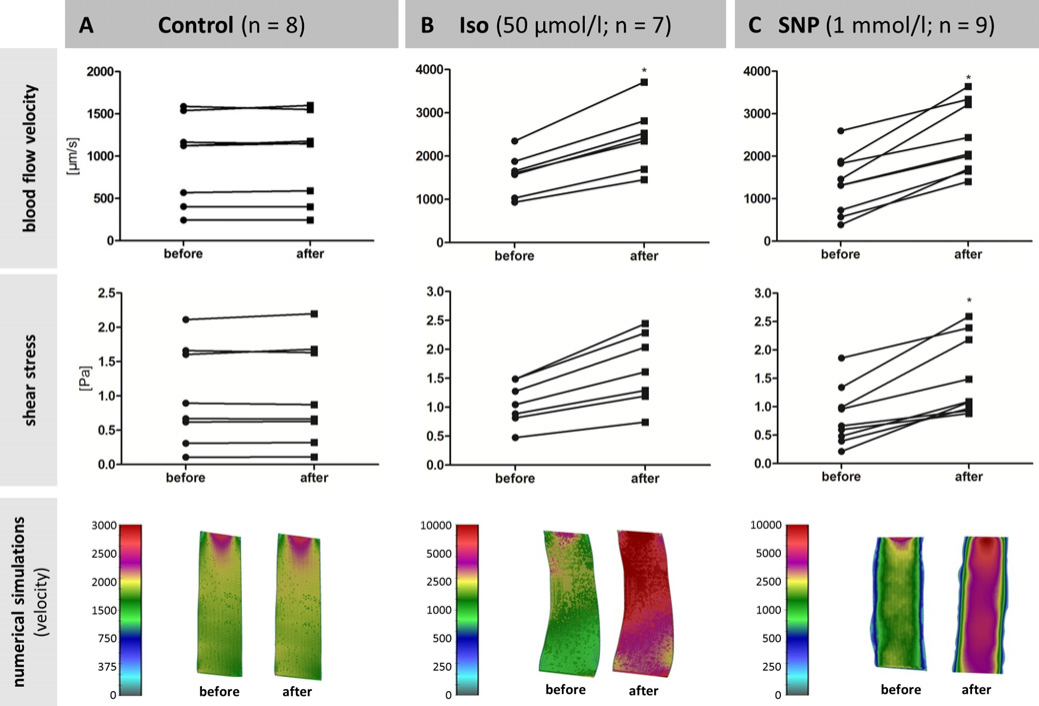
Hemodynamic response in the submental vein to application of 50 μmol/L Iso and 1 mmol/L SNP. Blood flow velocity, shear stress and an example of a numerical simulation of (A) control animals, (B) isoprenaline-treated, and (C) SNP-treated animals. Control fish were observed after 10 min of oral perfusion with system water (“before”) and 10 min later (“after”).

Blood flow velocity and shear stress in the submental vein of the control group remained constant from t_0_ (see Fig 5A: “before”) to t_10_ (see Fig 5A: “after”), even though the baseline values differed substantially (S2 Fig). The flow profile of the numerical simulations was coherent for both time points (Fig 5A). Application of 50 μmol/L Iso increased the blood flow velocity by 54.43 ± 1.21% *(n = 7*, *p = 0*.*026)* and shear stress by 54.23 ± 1.40% *(n = 7*, *p = 0*.*053)*. The graphical representation of the flow profile indicated an overall increase of blood flow velocity (Fig 5B). The effects of SNP on hemodynamic parameters at the level of the submental vein were investigated before and 10 min after treatment with the drug. Application of 1 mmol/L SNP with the oral perfusate significantly increased the blood flow velocity in all fish by 110.46 ± 19.64% *(n = 9*, *p = 0*.*013*; Fig 5C) while heart rates remained constant (Fig 3). In contrast to Iso, shear stress was also significantly affected by SNP (*p = 0*.*034*; Fig 5C). These results suggest that the use of NO-donors such as SNP is more potent to manipulate hemodynamic parameters in adult zebrafish than cardio-active agents, such as isoprenaline.

## Discussion

Using a new method to change cardiac and hemodynamic parameters in adult zebrafish *in vivo*, we show in this study that isoprenaline hydrochloride (Iso) increases the heart rate and blood flow velocity, and sodium nitroprusside (SNP) increases the blood flow velocity and shear stress in the submental vein. Together with the zebrafish’s excellent suitability for *in vivo*-microscopy and genetic modifications, the methodology described will be useful to study blood flow-dependent processes, such as angiogenesis. Such processes are well documented in embryonal and/or *in vitro*-models but need further attention in adult organisms.

In contrast to early zebrafish embryos, adult zebrafish are highly mobile and can microscopically only be studied under anesthesia. Although long-term investigations are possible [39], the routine use of tricaine (MS-222) for anesthesia is known to induce severe dose-dependent bradycardia [15]. This can partially be avoided by paralyzing the fish with an intraperitoneal injection of μ-conotoxin GIIIB (μ-CTX), an inhibitor of Na_v_ channels [21]. Together with an oral saltwater perfusion, this method was shown to be suitable for long-term cardiac investigations with the QT-interval remaining constant over several hours [21,22,23]. Other studies used the paralytic agents cisatracurium [40] or pancuronium [41], whereas in our case the paralytic agent μ-CTX [21] resulted in uniform paralysis. The frequency of pulsed oral perfusion was chosen to match the average gill-motion frequency of adult zebrafish [42].

Treatment with 50 μmol/L of Iso increased HR by 22.6 ± 1.3% after 20 min of treatment *in vivo*. The use of 500 μmol/L Iso did not further increase HR (20 ± 7.4%) and induced a higher HR variability. Therefore, we conclude that the use of 50 μmol/L Iso is adequate to produce maximal chronotropic stimulation. It has been previously reported that SNP may increase HR by stimulating the hyperpolarization-activated inward current in guinea pigs [43]. However, SNP did not change the HR in our experiments *in vivo*. Parameters for mathematical flow simulations like blood flow velocity and shear stress in the submental vein were obtained from *in vivo*-microscopic measurements. Several erythrocytes were tracked and the obtained values averaged. It is challenging to measure and keep track of the flow parameters in zebrafish blood vessels due to their small dimensions. The numerical simulations used in this study enable a general overview of the flow through the whole blood vessel segment. Fluid flow simulations can provide additional quantitative information about the state of the vessel (oxidized LDL-concentration in the wall [38], concentrations of macrophages and cytokines in the intima [33], diffusion coefficients, plaque growth [34] etc.). It can also be applied for analyzing the intravascular distribution and delivery of drugs [44] or for accurate modeling of erythrocyte deformation in complex geometrical domains [45]. However, this study mainly focused on blood flow velocity and shear stress.

Depending on the characteristic anatomy of individual zebrafish, the submental vein may be hidden, thus complicating imaging at high magnifications. Moreover, wrong placement of the 22G-perfusion needle may induce artifacts in blood flow by pinching the vein. Still, the submental vein is suitable to study changes in hemodynamic parameters. Our results were in accordance with a study conducted in humans, where SNP increased the blood flow by 86% [46]. It is likely that similar effects can be achieved in peripheral blood vessels other than the submental vein in the zebrafish, although further research needs to be conducted.

Taken together, we demonstrate how hemodynamics in the adult zebrafish model can be modulated by Iso and SNP. The findings form the foundation for investigating blood flow and shear stress-dependent processes such as intussusceptive angiogenesis also in adult zebrafish.

## Supporting Information

S1 Fig Detailed methodology used for blood flow simulations. (DOCX)

S2 Fig Examples of blood flow in the submental vein before and after treatment with 1 mmol/L sodium nitroprusside. (MP4)
