# Supplementary material for: Pharmacological Modulation of Hemodynamics in Adult Zebrafish In Vivo
Source: PLoS One. 2016 Mar 11;11(3):e0150948. doi: 10.1371/journal.pone.0150948 (PMC4788458; doi:10.1371/journal.pone.0150948)
Supplement: S1 Fig — (DOCX) [file pone.0150948.s001.docx]

**Supporting Information 1. Detailed methodology used for blood flow simulations.** Finite element mesh is generated using FEMAP software version 10 (Siemens PLM Software, USA), together with a custom-made software PakF [[31](#_ENREF_31),[32](#_ENREF_32)], which is then used to simulate blood flow. The three-dimensional flow of a viscous incompressible fluid is considered. The flow is governed by the Navier-Stokes equations and continuity equation that can be written as:

| $\rho\left(\frac{\partial v_{i}}{\partial t}+v_{j}\frac{\partial v_{i}}{\partial x_{j}}\right)=-\frac{\partial p}{\partial x_{i}}+\mu\left(\frac{\partial^{2}v_{i}}{\partial x_{j}\partial x_{j}}+\frac{\partial^{2}v_{j}}{\partial x_{j}\partial x_{i}}\right)$ | (1) |
| --- | --- |

| $\frac{\partial v_{i}}{\partial x_{i}}=0$ | (2) |
| --- | --- |

where v_i_ is the blood velocity in direction x_i_, ρ is the fluid density, p is pressure, μ is the dynamic viscosity; and summation is assumed on the repeated (dummy) indices, i,j=1,2,3. The first equation represents balance of linear momentum, while the equation (2) expresses incompressibility condition.

The wall shear stress is calculated as

|  | (3) |
| --- | --- |

where $\vec{v}_{t}$ denotes the tangential velocity close to the walls, and $\vec{n}$ is the normal direction at the vessel wall.

Solving the equations (1) and (2) the velocity field is obtained. Then using the tangential velocity near the wall surface, it is possible to numerically evaluate the velocity gradient . This way, 3 components $\left(\tau_{x},\tau_{y},\tau_{z}\right)$ of the wall shear stress vector $\vec{\tau}$ are obtained. The effective value of the wall shear stress $\tau_{eff}$ is calculated as:

| $\tau_{eff}=\sqrt{\tau_{x}^{2}+\tau_{y}^{2}+\tau_{z}^{2}}$ | (4) |
| --- | --- |

The boundary conditions for the simulation are set such that the velocity at the walls of the blood vessel is equal to zero. The initial conditions are defined such that the velocity at the inlet of the blood vessel is prescribed. The prescribed value of the velocity is calculated using experimental data. Imaging techniques are used to track individual red blood cells moving in the center of the diameter of the blood vessel and then information obtained this way is used to determine the velocity at the inlet of the considered blood vessel.

In the simulations, the units for physical dimensions of the domain are micrometers, obtained velocity distribution is shown in micrometers/second, obtained pressure and wall shear stress distributions are shown in Pascal (N/m^2^). Density of blood is set to 1.05 g/cm^3^, dynamic viscosity of blood is equal to $3.675\cdot10^{-3}\text{Pa}\cdot\text{s}$.
